# Assessment of free light chains in the cerebrospinal fluid of patients with lymphomatous meningitis – a pilot study

**DOI:** 10.1186/1471-2407-7-185

**Published:** 2007-10-03

**Authors:** B Hildebrandt, C Müller, A Pezzutto, PT Daniel, B Dörken, C Scholz

**Affiliations:** 1 Charité-Centrum für Tumormedizin, Medizinische Klinik mit Schwerpunkt Hämatologie und Onkologie, Campus Virchow-Klinikum, Charité- Universitätsmedizin Berlin, Augustenburger Platz 1, D-13344 Berlin, Germany; 2 Zentrum für Diagnostische und Präventive Labormedizin, Zentralinstitut für Laboratoriumsmedizin und Pathobiochemie, Campus Virchow-Klinikum, Charité-Universitätsmedizin Berlin, Augustenburger Platz 1, D-13344 Berlin, Germany

## Abstract

**Background:**

Lymphomatous meningitis (LM) represents a severe complication of malignant lymphomas. While clinical suspicion is raised by symptoms ranging from mild disturbances of sensation to severe pain or impaired consciousness, the definite diagnosis of LM is often difficult to obtain. Since B-cell lymphomas are clonally restricted to express either kappa or lambda immunoglobulin light chain, we hypothesised that analysis of free light chain (FLC) ratios might facilitate the diagnosis of LM.

**Methods:**

Kappa and lambda FLC were measured using a novel nephelometric assay in cerebrospinal fluid (CSF) and serum from 17 patients. 5/17 suffered from LM as demonstrated by cytology, immunocytology, and/or imaging procedures.

**Results:**

Measurement of FLC concentrations in CSF was achieved for all 17 patients. FLC levels in CSF were lower than serum FLC levels in samples for the same patient obtained at the same time (p < 0.01). CSF and serum FLC concentrations correlated weakly in all patients irrespective of LM status. Significantly more patients with cytopathologically and immunohistochemically proven LM displayed abnormal kappa/lambda FLC ratios in CSF compared to individuals with no LM (p < 0.01).

**Conclusion:**

This is the first report demonstrating that a significant proportion of LM patients display an abnormal kappa/lambda FLC ratio in the CSF.

## Background

Systemic intermediate- and high-grade Non-Hodgkin's Lymphomas (NHL) carry a substantial risk of central nervous system (CNS) complications, and are sometimes accompanied by lymphomatous meningitis (LM) [1-4]. Cytopathologic assessment of cerebrospinal fluid (CSF) represents the standard diagnostic procedure. Current imaging technologies have low sensitivity and specificity with respect to LM [5]. To increase the sensitivity of screening protocols for LM, multiple CSF punctures or immunocytology may be performed. However, normal CSF counts and/or normal CSF cell morphology do not rule out LM [3].

Assessment of free immunoglobulin light chains in CSF may improve the diagnostic investigation of LM. While most immunoglobulin light chains are bound to immunoglobulin heavy chains to form complete antibody molecules, excess light chains are produced and secreted as free light chains (FLC). Kappa and lambda FLC can be measured quantitatively in serum and urine using a novel nephelometric immunoassay. In normal individuals, serum concentrations of kappa FLC and lambda FLC range from 3.3 to 19.4 mg/l and 5.7 to 26.3 mg/l, respectively. Serum FLC ratios (kappa/lambda) lying outside the normal range (0.26 to 1.65, [6]) indicate monoclonal FLC production arising as a result of clonal expansion [6,7]. Serum FLC levels and ratios are now routinely measured to diagnose and monitor disease progression and response to therapy in light chain multiple myeloma [8], nonsecretory myeloma [9], intact immunoglobulin myeloma [10,11] primary AL amyloidosis [12], and to risk stratify patients with monoclonal gammopathy of unknown significance (MGUS) [13]. B-cell Non-Hodgkin's Lymphomas likewise exhibit a light chain restriction, so that measuring free light chain concentrations in CSF could particularly aid the diagnosis of LM.

## Methods

### Patients

Between December 2005 and August 2006, we obtained CSF and peripheral blood samples from 17 consecutive patients with clinically suspicion of LM who underwent diagnostic lumbar punctures. All lumbar punctures were clinically indicated and patients gave their informed consent prior to CSF and peripheral blood sampling. For patient's characteristics refer to table 1.

**Table 1:** Patients' characteristics

**Patient**	**Gender**	**Age**	**LM**	**Diagnosis**	**CT/MRI**	**CSF cell count**	**CSF cytology**	**CSF-IC**	**Light chain on primary**
1	W	46	Yes	CLL	LM	1054	Lymphocytes	Kappa	Kappa
2	W	47	Yes	LPL	Focal lesions	25	Lymphocytes	Kappa	Kappa
3	M	64	Yes	DLCL, Stage IV	N.D.	109	Lymphoblasts	N.D.	N.D.
4	M	65	Yes	LPL	LM	113	Lymphocytes	N.D.	Kappa
5	M	68	Yes	DLCL, Stage IVE	NEM	24	Lymphocytes	Kappa	Kappa
6	M	28	No	PTLPD, Stage IVE	NEM	<10	N.S.C.	N.D.	N.D.
7	M	29	No	FL Stage Ie (tonsil)	NEM	<10	N.S.C.	N.D.	N.D.
8	M	37	No	DLCL Stage II	NEM	<10	N.S.C.	N.D.	N.D.
9	M	46	No	DLCL, Stage IVE	NEM	23	N.S.C.	N.D.	Kappa
10	W	50	No	DLCL, Stage III	Focal Lesion	<10	N.S.C.	N.D.	N.D.
11	M	63	No	M. Hodgkin, Stage IV	N.D.	<10	N.S.C.	N.D.	No
12	W	66	No	Intracerebral PTLPD	Focal Lesion	119	Lymphocytes	CD 3	N.D.
13	W	66	No	Lymphoblastic T-NHL	N.D.	<10	N.S.C.	N.D.	No
14	M	73	No	Ocular Lymphoma	NEM	<10	N.S.C.	N.D.	N.D.
15	M	77	No	CLL, Parkinson's Disease	NEM	<10	N.S.C.	N.D.	No
16	M	79	No	M. Hodgkin, Stage III	NEM	27	N.S.C.	N.D.	No
17	M	82	No	DLCL St. IE (Spinal Cord)	N.D.	90	Lymphocytes	CD3	N.D.

LM – lymphomatous meningitis; CT – computed tomography; MRI – magnetic resonance imaging; CSF – cerebrospinal fluid; IC – immuncytochemistry, CLL – chronic lymphocytic leukaemia; DLCL – diffuse large cell lymphoma; LPL – lymphoplasmocytic lymphoma, PTLPD – post-transplantation lymphoproliferative disorder; FL – follicular lymphoma; T-NHL – Non-Hodgkin's Lymphoma of T-cell origin.

NEM – no evidence of lymphomatous meningitis; N.S.C. – no suspicious cells; N.D. – not done; EP-4 – epithelial cell surface marker

### Routine CSF assessment

All CSF samples were routinely assessed for cell count, protein, glucose, and lactate content. Furthermore, microbiological and virological investigations were performed as per routine protocol for suspected bacterial or viral meningitis [14,15]. All CSF samples were also evaluated cytopathologically. For this purpose, 200 μl undiluted CSF was centrifuged using a Shandon Cytospin 3 centrifuge at 500 rpm for 5 minutes onto a glass slide. Samples were then stained according to the Pappenheim method. If dubious, additional cytospins were stained immunohistochemically according to the alkaline phosphatase-anti-alkaline phosphatase (APAAP) method with antibodies against the pan-B-cell antigen CD20, the kappa light chain, the lambda light chain, or the pan-T-cell marker CD3.

### Routine imaging

The majority of patients with suspected LM received a computed tomography and/or a magnetic resonance imaging of the brain as part of the diagnostic work-up prior to lumbar puncture.

### Free light chain assay

FLC analysis was performed using the serum free light chain assay (Freelite™ – The Binding Site Ltd., Birmingham, UK) on peripheral blood and CSF samples obtained at the same time. Freelite™ nephelometric assays [7] use latex-bound anti-FLC antibodies, reacted with serum/CSF samples on an automated BN Prospec nephelometer (Dade-Behring, Schwalbach, Germany). According to the manufacturer, the lower detection limit is 0.06 mg/l for kappa FLC and 0.08 mg/l for lambda FLC. We used 0.10 mg/l as the lower limit for both assays. In our institution, the inter-assay coefficients of variation (CV, n = 10) were 5.1% at 14.3 mg/l for kappa FLC and 3.6% at 28.1 mg/l for lambda FLC assay in control sera provided by the manufacturer. With these control sera, appropriately diluted for the quality control of CSF measurements [16], inter-assay CVs of 9.8% at 0.8 mg/l kappa FLC and 5.8% at 1.5 mg/l lambda FLC were determined.

### Statistical analyses

Descriptive and comparative statistics were performed by using the SPSS 12 software package. Mann-Whitney-tests were applied for comparisons between groups. Correlations were assessed by using Kendall-Tau-b correlation coefficients. A two-sided p-value less than 0.05 was considered to indicate significance for all tests. Sensitivity and specificity were calculated by using 2 × 2 tables on the basis of the upper reference values of the respective light chain serum values.

## Results

### Patients characteristics

Between 12/2005 and 08/2006 peripheral blood and CSF samples from 17 patients with lymphomas were assessed. 14/17 experienced clinical symptoms highly suspicious of LM and/or displayed suspect findings on imaging. In the remaining 3 patients, lumbar puncture was indicated according to recent diagnostic guidelines. Those comprised one ocular high-grade B-cell Non-Hodgkin's Lymphoma (B-NHL), one B-NHL with a paravertebral manifestation progressing into the spinal canal, and one high-grade follicular lymphoma of the tonsil (Table 1).

By means of cytopathology and immunohistochemistry, LM was diagnosed in 5 patients. The other 12 patients demonstrated no definite evidence indicating LM following routine assessment.

Patient characteristics, results from conventional CSF diagnostics with/without immunohistochemistry, and findings from CNS imaging are presented in table 1.

### Comparison of free light-chain concentrations in CSF and serum

Kappa and lambda FLC were quantified in serum and CSF for each patient (Table 2). Both kappa FLC and lambda FLC levels were significantly higher in serum than in CSF (p < 0.01) in patients with and without LM. In addition, the range of kappa FLC levels was wider in serum than in CSF. The range of CSF FLC ratios was found to be narrower than the range of serum FLC ratios, but overall there was no significant difference between the ratios in CSF and serum (p = 0.53). Correlation coefficients between CSF and serum samples revealed weak to moderate correlations for kappa and lambda FLC (+0.47; +0.26) as well as for the kappa/lambda FLC ratio (+0.51) (Table 3).

**Table 2:** Free light chain concentrations in CSF and serum *

**Patient**	**LM**	**CSF-κ**	**CSF-λ**	**CSF-Ratio**	**Serum-κ**	**Serum-λ**	**Serum-Ratio**
1	Yes	30.1	1.4	21.3	41.9	1.6	26.2
2	Yes	6.4	0.4	15.0	13.3	1.2	11.5
3	Yes	3.0	0.8	3.8	14.4	17.4	0.8
4	Yes	5.3	0.1	53.0	868.0	0.7	1240.0
5	Yes	32.6	0.4	85.8	114.0	13.5	8.4
6	No	0.2	0.1	1.9	15.2	17.3	0.9
7	No	0.4	0.4	1.0	5.6	12.4	0.5
8	No	0.5	0.4	1.2	28.1	1.7	16.7
9	No	16.9	4.8	3.6	41.2	32.3	1.3
10	No	18.5	16.4	1.1	18.5	16.4	1.1
11	No	0.4	0.4	0.9	26.9	35.9	0.7
12	No	11.5	2.8	4.0	30.1	19.7	1.5
13	No	0.7	0.4	1.6	5.6	1.6	3.5
14	No	0.3	0.4	0.7	N.D.	N.D.	N.D.
15	No	0.3	0.7	0.4	8.9	11.4	0.8
16	No	10.2	2.7	3.7	39.3	33.1	1.2
17	No	0.5	0.4	1.2	18.6	15.6	1.2

* values for kappa and lambda given in mg/l.

LM – lymphomatous meningitis; CSF – cerebrospinal fluid; N.D. – not done

**Table 3:** Comparison of light chain concentrations in cerebrospinal fluid and serum in 17 patients

	**κ CSF**	**κ Serum**	**λ CSF**	**λ Serum**	**κ/λ CSF**	**κ/λ Serum**
Reference (mg/l)	not defned	3.30–19.40	not defined	5.71–26.3	not defined	0.26–1.65
Range (mg/l)	0.2 – 32.6	5.6 – 868.0	0.1 – 16.4	0.7 – 35.9	0.4 – 85.8	0.5 – 1240.0
Mean ± *SD*	8.1 ± 10.1	80.6 ± 211.6	1.9 ± 3.9	14.5 ± 11.7	12.1 ± 23.9	82.3 ± 308.8
Correlation	+ 0.47		+ 0.26		+ 0.51	
P for Difference	< 0.01		< 0.01		0.53	

### Comparison of FLC concentrations between patients with and without LM

**Figure 1:**
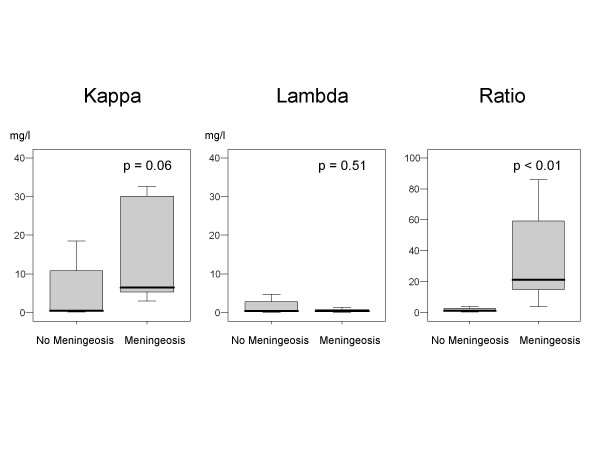
Comparison of kappa FLC, lambda FLC, and kappa/lambda FLC ratios in patients with and without lymphomatous meningitis.

We analysed CSF FLC concentrations in patients with and without proven LM. Two patients with LM presented kappa FLC levels in CSF greater than the upper limit of the normal serum reference range. By contrast, patients without LM displayed kappa FLC levels in CSF lower than the upper limit of the normal serum reference range. Further, the mean kappa FLC levels in CSF were approximately threefold higher in patients with LM compared to those without LM (15.5 mg/l vs, 5.0 mg/l; p = 0.06).  Also see figure 1.

It is of note that no elevations of lambda FLC levels in CSF were detected within the entire study population.

8/12 individuals without LM exhibited CSF kappa/lambda FLC ratios within the serum reference range (0.26 to 1.65). In contrast, all (5/5) of the patients with proven LM demonstrated CSF kappa/lambda FLC ratios outside the serum reference range. The CSF kappa/lambda FLC ratios in patients with and without LM was significantly different (p < 0.01). Assessment of the sensitivity and specificity of CSF kappa/lambda FLC ratio for the diagnosis of LM resulted in a sensitivity of 100% and a specificity of 67% (Table 4).

**Table 4:** Comparison of CSF free light chain concentrations in patients without (n = 12) and with (n = 5) lymphomatous meningitis

	**κ without**	**κ with**	**λ without**	**λ with**	**κ/λ without**	**κ/λ with**
Range (mg/l)	0.2 – 18.5	3.0 – 32.6	0.1 – 16.4	0.1 – 1.4	0.4 – 4.1	3.8 – 85.8
Mean ± SD	5.0 ± 7.2	15.5 ± 14,6	2.5 ± 4.6	0.6 ± 0.5	1.8 ± 1.3	37.0 ± 34.3
Values lower/higher NSV	8/0	1/2	11/0	5/0	0/4	0/5
Sensitivity (> UNSR)	0.40		0.00		1.00	
Specifity (> UNSR)	1.00		1.00		0.67	

NSR – normal of serum reference

UNSR – Upper normal serum reference

Clinical details of the individuals without proven LM but with CSF kappa/lambda FLC ratios outside the serum reference range (4/12) were as follows: Patient 12 (CSF kappa/lambda ratio of 4 (Table 2) had an intracerebral manifestation of a post-transplantation lymphoproliferative disorder. While the CSF-FLC ratio suggested possible LM, this was not supported by conventional diagnostics. The patient died a few weeks after the initial lumbar puncture due to disease progression. Patient 9 (CSF kappa/lambda FLC ratio of 3.6) had clinical symptoms highly suggestive of central nervous system (CNS) involvement and lesions reminiscent of a stroke after CNS imaging. Clinical symptoms quickly improved after empirical intrathecal therapy with methotrexate combined with systemic chemoimmunotherapy. Remarkably, the CSF kappa/lambda FLC ratio decreased from 3.6 to 1.6, while the serum kappa/lambda FLC ratio remained largely unchanged at a value of 1.63 (data not shown), and subsequent imaging of the brain displayed a recurrence of the former lesions. Patient 6 (CSF kappa/lambda FLC ratio of 1.9) suffered from a post-transplantation lymphoma without cerebral manifestation. Finally patient 16 (CSF kappa/lambda FLC ratio of 3.7) had an Epstein Barr virus-associated B-cell lineage Hodgkin's disease.

## Discussion

Lymphomatous meningitis is a typical, but uncommon manifestation of systemic lymphoma or primary CNS lymphoma. To date, the diagnosis is generally obtained through cytopathologic assessment of CSF. However, normal CSF cell counts do not rule out the presence of LM and even repeated CSF examinations, immunohistochemistry, or flow cytometry do not generally enhance diagnostic specificity [17-19].

To evaluate the benefit of measuring FLC concentrations, and the kappa/lambda FLC ratio, in the CSF for the diagnosis of LM, we performed a pilot study measuring FLC in serum and CSF from 17 patients with lymphomas.

The data presented here demonstrates that quantification of absolute FLC concentrations in CSF is possible in lymphoma patients. Interestingly, CSF FLC levels were significantly lower compared to serum FLC levels. This observation might be explained due to the fact that normal CSF is largely devoid of FLC-producing B-cells, and that only small amounts of immunoglobulin cross the blood-brain barrier [20]. Supporting this suggestion, patient 8 who displayed an abnormal serum kappa/lambda FLC ratio of 16.7, but a normal CSF FLC ratio, had a relapsed diffuse large cell lymphoma, a leachate of lymphoma cells into peripheral blood, and a large abdominal lymphoma burden. This patient therefore had a potentially disrupted blood-brain barrier.

A recent publication reported CSF FLC kappa levels in the range 0.06 to 0.47 mg/l in patients with no oligoclonal immunoglobulin banding after electrophoresis, undergoing a routine CSF assessment [16]. For the patients in our study without definite LM, and with a normal FLC kappa/lambda ratio (8/12), the range was 0.3 to 18.5 mg/l. However, if the patient with a concentration of 18.5 mg/l is excluded, the range for the remaining 7/8 patients was between 0.3 and 0.7 mg/l. Thus, it is very likely that the normal range of FLC concentrations in CSF is generally below that reported in serum. These normal reference ranges need to be confirmed by larger studies.

Of particular interest, we found that when comparing CSF FLC ratios in patients with and without conventionally proven LM, we detected significantly more patients with abnormal CSF FLC kappa/lambda ratios (p < 0.01) in the confirmed LM group. Indeed, all patients (5/5) with proven LM exhibited abnormal CSF kappa/lambda FLC ratios, while most (8/12) patients without definite LM demonstrated normal CSF kappa/lambda ratios. It is of note, however, that 2/4 patients (patient 9 and 12) with abnormal CSF FLC ratios but without LM had definite or very likely intracerebral lymphoma manifestations. LM is far more likely to occur in these patients than in those with systemic NHL without parenchymal involvement of the brain.

Finally, it is of note that in our pilot study all patients with definite LM exhibited a kappa light chain restriction. Twice as many B cells in normal peripheral blood produce kappa FLC as produce lambda FLC. It is likely that this ratio will be reflected in there being twice as many B cell NHL producing kappa FLC as lambda FLC. This production ratio and the small number of patients included in this pilot study might explain why we only detected LM in NHL patients with a kappa light chain restriction. The utility of the CSF kappa/lambda FLC ratio for the detection of LM in NHL patients with a lambda light chain restriction will be the subject of a future study involving a larger number of patients.

## Conclusion

In conclusion, we demonstrate for the first time that the CSF kappa/lambda FLC ratio appears to be a promising parameter for use in diagnosing lymphomatous meningitis. Further research, including the confirmation of these findings in a larger cohort of patients, might establish CSF kappa and lambda FLC analysis as a novel diagnostic tool for patients with suspected lymphomatous meningitis.

## Abbreviations

CNS, central nervous system; CSF, cerebrospinal fluid; FLC, free light chain; LM, lymphomatous meningitis.

## Competing interests

The author(s) declare that they have no competing interests.

## Authors' contributions

BH co-developed the idea of the study, was involved into the provision of patients, performed the statistics and participated in writing the manuscript. CM measured the samples and participated in writing the manuscript. AP was involved into the provision of patients, gave administrative support and participated in writing the manuscript. PTD was involved into the provision of patients, gave administrative support, and participated in writing the manuscript. BD: was involved into the provision of patients, gave administrative support, and participated in writing the manuscript. CS: co-developed the idea of the study, was involved into the provision of patients, and wrote the first draft of the manuscript. All authors read and approved the final manuscript.

## Pre-publication history

The pre-publication history for this paper can be accessed here:


